# AP-1-Targeted Anti-Inflammatory Activities of the Nanostructured, Self-Assembling S5 Peptide

**DOI:** 10.1155/2015/451957

**Published:** 2015-05-12

**Authors:** Woo Seok Yang, Young-Jin Son, Mi-Yeon Kim, Soochan Kim, Jong-Hoon Kim, Jae Youl Cho

**Affiliations:** ^1^Department of Genetic Engineering, Sungkyunkwan University, Suwon 440-746, Republic of Korea; ^2^Department of Pharmacy, Sunchon National University, Suncheon 540-742, Republic of Korea; ^3^School of Systems Biological Science, Soongsil University, Seoul 156-743, Republic of Korea; ^4^Department of Veterinary Physiology, College of Veterinary Medicine, Biosafety Research Institute, Chonbuk National University, Jeonju 561-756, Republic of Korea

## Abstract

Peptide-based therapeutics have received increasing attention in medical research. However, the local delivery of such therapeutics poses unique challenges. Self-assembling peptides that use decorated nanofibers are one approach by which these therapeutics may be delivered. We previously found that the self-assembling K5 peptide affects the anti-inflammatory response. The aim of the present study was to investigate another self-assembling peptide, S5. Unlike the K5 peptide which has a positive charge, the S5 peptide has a free hydroxyl (-OH) group. We first examined whether the S5 peptide regulates the inflammatory response in primary cells and found that the S5 peptide reduced the production of prostaglandin E_2_ (PGE_2_) and tumor necrosis factor (TNF)-*α* in lipopolysaccharide- (LPS-) treated bone marrow-derived macrophages. Moreover, the S5 peptide significantly downregulated *cyclooxygenase- (COX-) 2, TNF-α*, and *interleukin- (IL-) 1β* expression by blocking the nuclear translocation of c-Jun. Consistent with this finding, the S5 peptide diminished the activation of inflammatory signaling enzymes related to p38. The S5 peptide also inhibited the formation of the p38/c-Jun signaling complex in RAW264.7 cells. Similarly, p38 and MKK3/6 were inhibited by the S5 peptide in LPS-activated peritoneal macrophages. Taken together, these results strongly suggest that the S5 peptide could exert anti-inflammatory effects by inhibiting the c-Jun/p38 signaling pathway.

## 1. Introduction

Innate immune responses provide four anatomical, physiological, phagocytic, and inflammatory barriers. Among these barriers, the macrophage-mediated inflammatory barrier is the most important [1]. Macrophages produce proinflammatory cytokines and other signaling molecules such as interleukins (ILs), tumor necrosis factor (TNF)-*α*, and prostaglandins (PGs), all of which are essential for the initial inflammatory response [2]. Thus, the initial activation of macrophages is a significant event in inflammatory processes. To activate the inflammatory response, macrophage surface receptors such as Toll-like receptors (TLRs) interact with their cognate ligands [3]. Activated macrophages consequently upregulate intracellular signaling pathways to activate the nuclear factor (NF)-*κ*B pathway and the activator protein- (AP-) 1 pathway, leading to the expression of inflammatory genes [4–6]. To cure inflammatory diseases, effective regulation of the inflammatory response is required. Therefore, targeted inhibition of intracellular inflammatory-related signaling pathways has been considered to be a promising therapeutic strategy for treating various immunological diseases such as cancer, septic shock, diabetes, and atherosclerosis [7–11]. The applications of self-assembling peptides have been widely investigated. Biological hydrogels consisting of self-assembling peptide nanofibers have been shown to have a broad range of potential applications [12–15]. Self-assembling peptides consist of short peptides with 8 to 16 residues that are 2.5 to 5 nm in length, depending on conditions such as pH and ionic strength [16]. These structural features have made self-assembling systems an attractive option for tissue culture-based and tissue-based research into the mechanisms that control various cellular processes [17]. Although recent studies have indicated that self-assembling peptides have potential applications in drug delivery as a nanomedicine platform [18], the molecular mechanisms underlying these applications are not yet fully understood.

In our previous study, we determined the immune-regulatory functions of a self-assembling peptide, K5, RADARADARADARADA-KKKKK. In the present study, we investigated another peptide named S5, RADARADARADARADA-SSSSS, with 5 serine resides, which was developed to investigate polarity effects on self-assembling property, plasma protein interactions, and platelet activation [19]. While the K5 peptide exhibits lysine residue-driven net positive charge at a pH of 7.4, disadvantageous in terms of membrane permeability, S5 peptide only maintains free hydroxyl group. Considering chemical property of S5, it is hypothesized that the penetration of S5 into cytoplasmic compartment in a cell might be better than K5. In this study, we aimed to demonstrate the immunopharmacological improvement of the S5 peptide in cellular and molecular inflammatory responses.

## 2. Materials and Methods

### 2.1. Materials

The S5 peptide was purchased from SynBioSci (Livermore, CA, USA). Tetrazole (3-4,5-dimethylthiazol-2-yl)-2,5-diphenyltetrazolium bromide (MTT), prednisolone and indomethacin, and lipopolysaccharide (LPS,* E. coli* 0111:B4) were purchased from Sigma Chemical Co. (St. Louis, MO, USA). Enzyme immunoassay (EIA) kits and enzyme-linked immunosorbent assay (ELISA) kits for determining the levels of PGE_2_ and TNF-*α* were purchased from Thermo Scientific (NYSE: TMO) and Amersham (Little Chalfont, Buckinghamshire, UK). Fetal bovine serum (FBS), RPMI1640 and DMEM media were purchased from Thermo Scientific (NYSE: TMO). RAW264.7 and HEK293 cells were obtained from ATCC (Rockville, MD, USA). Phospho- and total protein antibodies against c-Jun, extracellular signal-related kinase (ERK), c-Jun N-terminal kinase (JNK), p38, MAP kinase kinase 3/6 (MKK3/6), TGF-*β* activated kinase 1 (TAK1), lamin A/C, and *β*-actin were purchased from Cell Signaling (Beverly, MA, USA) and Santa Cruz (Santa Cruz, CA, USA).

### 2.2. Animals

Male ICR mice (6 weeks old, 17 to 21 g) were purchased from DAEHAN BIOLINK (Chungbuk, Korea) and housed in plastic cages under conventional conditions. Water and food pellets (Samyang, Daejeon, Korea) were supplied* ad libitum*. All studies were carried out in accordance with the guidelines established by the Institutional Animal Care and Use Committee of Sungkyunkwan University (approval ID: SKKUBBI 12-6-2).

### 2.3. Preparation of Bone Marrow-Derived Macrophages

Bone marrow- (BM-) derived macrophages were obtained from ICR male mice (6 weeks old, 17 to 21 g) by severing the bone of the femoral region. Bone marrow-derived macrophages were flushed out into Petri dishes with PBS. After harvesting bone marrow cells by centrifugation at 1,200 rpm for 5 minutes at 4°C, the cells were resuspended in RPMI1640 medium containing 5% FBS. Finally, bone marrow-derived macrophages (1 × 10^6^ cells/mL) were seeded in 100 mm tissue culture dishes and grown for 4 h at 37°C in a humidified atmosphere with 5% CO_2_.

### 2.4. Cell Culture

Primary cells (bone marrow-derived macrophages) and cell lines (RAW264.7 and HEK293 cells) were cultured with RPMI1640 medium supplemented with 10% heat-inactivated FBS, glutamine, penicillin, and streptomycin at 37°C under 5% CO_2_.

### 2.5. Determination of PGE_2_ and TNF-*α* Production

RAW264.7 cells and bone marrow-derived macrophages (1 × 10^6^ cells/mL) were preincubated for 18 h, after which cells were treated with the S5 peptide (0 to 100 *μ*g/mL) or standard compounds (prednisolone and indomethacin) for 30 min and then additionally treated with LPS (1 *μ*g/mL) for 24 h. Production and the release of PGE_2_ and TNF-*α* were determined by EIA and ELISA kits, as described previously [20].

### 2.6. Cell Viability Assay

Bone marrow-derived macrophages or RAW264.7 cells (1 × 10^6^ cells/mL) were treated with S5 peptide (0 to 100 *μ*g/mL) for 24 h. Cytotoxic effect of S5 peptide was evaluated by conventional MTT assay as described previously [21, 22].

### 2.7. mRNA Expression Analysis by Quantitative Reverse Transcriptase-Polymerase Chain Reaction

To evaluate inflammatory gene mRNA expression levels, RAW264.7 cells pretreated with S5 peptide (0 to 100 *μ*g/mL) for 30 min were incubated with LPS (1 *μ*g/mL) for 6 h. Total RNA from the cells was then isolated with TRIzol Reagent (Gibco BRL) according to the manufacturer's instructions and stored at −70°C until use. The levels of* IL-1β*,* TNF-α*, and* COX-2* mRNA expression levels were quantified by real-time reverse transcriptase-polymerase chain reaction (RT-PCR) with SYBR Premix Ex Taq according to the manufacturer's instructions (Takara, Japan). Reactions were run in a real-time thermal cycler (Bio-Rad, USA), as reported previously [23, 24]. Results are expressed as relative ratios of gene expression normalized to the internal control GAPDH. Primers were ordered from Bioneer (Seoul, Korea) and their sequences are listed in Table 1.

### 2.8. Preparation of Total Lysates and Nuclear Extracts, Immunoblotting, and Immunoprecipitation

Preparation of total lysates and nuclear extracts from LPS-treated RAW264.7 cells pretreated with S5 peptide was used, a method previously published [25]. Immunoblotting analysis of phosphorylated and total protein [c-Jun, lamin A/C, MAPK (ERK, p38, and JNK), MKK3/6, MEK1/2, MKK4/7, MKK4, TAK1, lamin A/C, and *β*-actin] levels was performed, according to published methods [26]. For immunoprecipitations, cell lysates were generated from RAW264.7 cells (1 × 10^7^ cells/mL) that had been treated either with or without LPS (1 *μ*g/mL) for 90 min. Lysates containing equal amounts of protein (500 *μ*g) were precleared with 10 *μ*L protein A-coupled Sepharose beads (50% v/v) (Amersham, UK) for 1 h at 4°C. Precleared samples were incubated overnight at 4°C with 5 *μ*L antibody to p38. Immune complexes were captured with 10 *μ*L protein A-coupled Sepharose beads (50% v/v) by rotation for 3 h at 4°C.

### 2.9. Statistical Analysis

**Figure 1:**
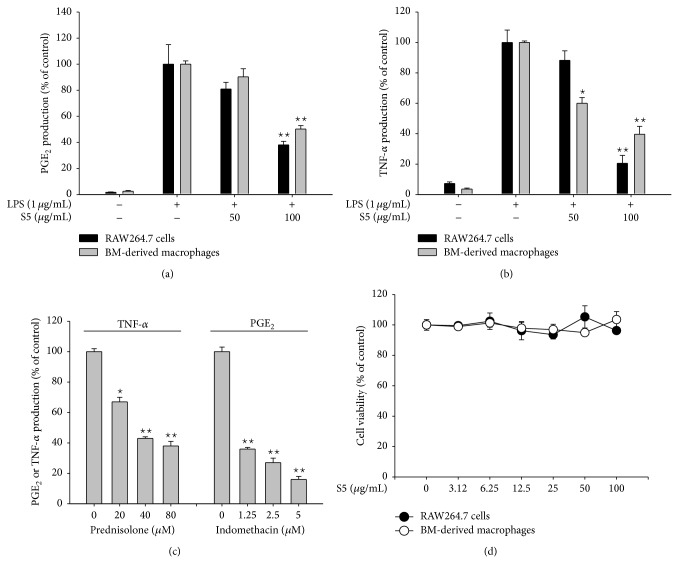
Effects of the S5 peptide on TNF-*α* and PGE_2_ production and viability of LPS-treated RAW264.7 cells and BM-derived macrophages. (a, b, and c) The levels of NO, PGE_2_, and TNF-*α* in the supernatants of RAW264.7 cells or bone marrow-derived macrophages that had been treated with the S5 peptide (0 to 100 *μ*g/mL) or standard compounds (prednisolone and indomethacin) in the presence or absence of LPS (1 *μ*g/mL) for 6 (TNF-*α*) or 24 (PGE_2_) h were analyzed by EIA and ELISA, respectively. (d) The viability of RAW264.7 cells and BW-derived macrophages was determined by the MTT assay. ^∗^ *P* < 0.05 and ^∗∗^ *P* < 0.01 compared with control cells; ^∗^ *P* < 0.05 compared with normal cells.

**Figure 2:**
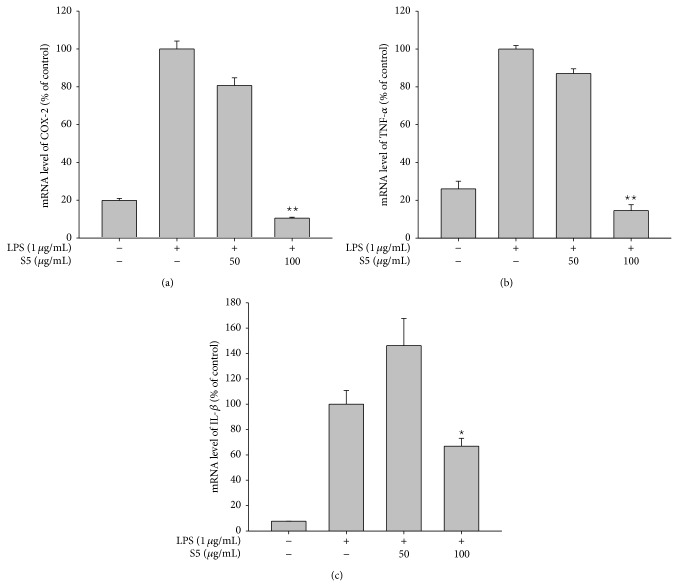
Effects of the S5 peptide on the mRNA expression levels of proinflammatory genes. The mRNA levels of TNF-*α*, COX-2, and IL-1*β* in RAW264.7 cells that had been pretreated with the S5 peptide and stimulated with LPS (1 *μ*g/mL) for 6 h were determined by real-time PCR. ^∗^ *P* < 0.05 compared with control cells.

Data (Figures 1 and 2) are expressed as means ± standard deviations (SDs) and were calculated from one of two independent experiments. Each experiment was performed with six technical replicates. The data shown in this paper are representative of three independent experiments, each of which yielded similar results. For statistical comparisons, results were analyzed using analysis of variance/Scheffe's post hoc test and the Kruskal-Wallis/Mann-Whitney tests. All *P* values < 0.05 were considered statistically significant. All statistical tests were carried out using the computer program SPSS (SPSS Inc., Chicago, IL, USA).

## 3. Results and Discussion

We previously reported that the self-assembling K5 peptide, applied as 3D scaffolds for tissue engineering or drug delivery [18, 27], downregulates the macrophage-mediated inflammatory responses [28]. The S5 peptide is part of a family of self-assembling peptides that includes the K5 peptide. However, in contrast to the K5 peptide, the S5 peptide lacks positive charge in this backbone. Since these two peptides exhibit reasonably similar structures with only a minor difference, we hypothesized that the S5 peptide would also exert anti-inflammatory effects.

Similar to the pharmacological profiles of K5 peptide [28], the S5 peptide appears to be a promising therapeutic molecule for dampening the inflammatory response. For instance, the S5 peptide decreased the production of PGE_2_ (Figure 1(a)) and TNF-*α* (Figure 1(b)) in both malignant RAW264.7 cells and in BM-derived LPS-activated macrophages in a dose-dependent manner as seen in the cases of standard compounds prednisolone and indomethacin (Figure 1(c)); importantly, no cytotoxicity was observed in these cells (Figure 1(d)). These data raised a possibility that S5 peptide can be a peptide drug with anti-inflammatory property as previously suggested with synthetic TIP-like peptide AP318, peptides from bovine *β*-casein, cathelicidin, and N15P polypeptide [29–31]. Since elucidating the molecular mechanisms of these effects is important for initiation of drug discovery process, we next examined the anti-inflammatory mechanism of the S5 peptide.

**Figure 3:**
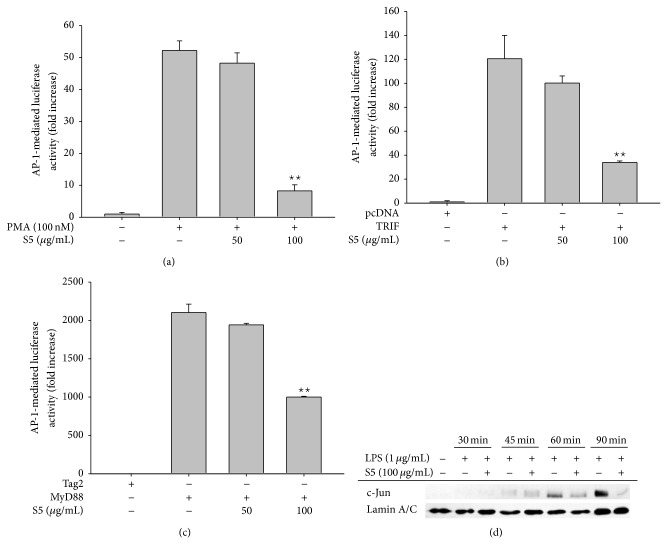
Effects of the S5 peptide on the activation of transcription factors. (a, b, and c) HEK293 cells that had been cotransfected with constructs driving the expression of AP-1-Luc, TLR adaptor molecules (MyD88 and TRIF), and *β*-gal (as a control) were treated with the S5 peptide (0 to 100 *μ*g/mL) in the presence or absence of PMA (100 nM). The resultant luciferase activity was measured with a luminometer. (d) The nuclear translocation level of a transcription factor in the AP-1 family (c-Jun) was assessed by immunoblotting analysis with antibodies against total c-Jun. ^∗^ *P* < 0.05 and ^∗∗^ *P* < 0.01 compared with control cells.

To examine the effects of the S5 peptide on the inflammatory response on the transcriptional level, the mRNA levels of* COX-2* (Figure 2(a)),* TNF-α* (Figure 2(b)), and* IL-1β* (Figure 2(c)) were measured by real-time PCR in cells that had been stimulated with LPS. These experiments showed that the S5 peptide (100 *μ*g/mL) downregulated the expression of* COX-2*,* TNF-α*, and* IL-1β*. To investigate whether the S5 peptide regulates DNA promoter activity, a luciferase reporter assay was used that resulted in cell luminescence. The S5 peptide remarkably decreased AP-1-mediated transcriptional activation in response to PMA, MyD88, or TRIF; moreover, this effect was dose-dependent (Figures 3(a), 3(b), and 3(c)). Activation of the inflammatory response is known to result in nuclear translocation of the AP-1 transcription factor, which results in transcription of AP-1-driven target genes.

**Figure 4:**
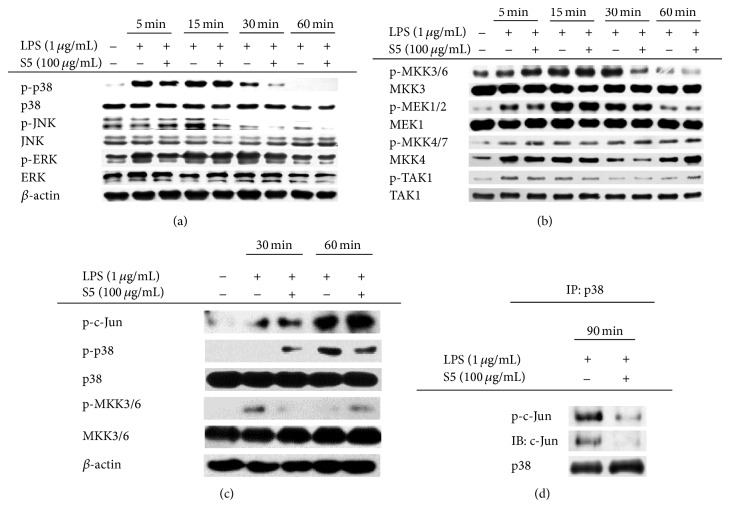
Effects of the S5 peptide on the activation of the AP-1 signaling pathway. (a and b) Phosphoprotein and total protein levels of p38, JNK, ERK, MEK1/2, MKK3/6, MKK4/7, TAK1, and *β*-actin in the lysates of RAW264.7 cells were analyzed by Western blotting. (c) The levels of total and phosphorylated c-Jun, p38, MKK3/6, and *β*-actin in lysates from peritoneal macrophages were determined by immunoblotting analysis. (d) The effect of the S5 peptide on the c-Jun/p38 interaction was determined by immunoprecipitation analysis with antibody against p38 and by immunoblotting analysis with antibodies against p-c-Jun, c-Jun, and p38.

To examine the nuclear translocation of c-Jun in the inflammatory response, we determined the levels of c-Jun in the nuclear fractions of LPS-treated RAW264.7 cells. This is because peptide K5 displayed predominant inhibitory activity on AP-1 translocation. Interestingly, these experiments showed that the S5 peptide prevented the nuclear translocation of c-Jun at 60 and 90 min (Figure 3(d)). This result implies that the S5 peptide may regulate the AP-1 signaling pathway. To investigate the activation status of upstream signaling molecules in the AP-1 pathway, the phosphorylation of various MAPKs (ERK, p38, and JNK) was analyzed by Western blotting. The phosphorylation of p38 and JNK was decreased at 30 and 15 min, while ERK phosphorylation was not decreased by the S5 peptide (Figure 4(a)). Interestingly, the S5 peptide only prevented phosphorylation of MKK3/6, an upstream kinase of p38 [32], whereas the S5 peptide did not prevent the phosphorylation of the upstream molecule JNK, which is downstream of MKK4/7. Phosphorylation of TAK1, a kinase that is upstream of the MKKs [33], was also decreased by the S5 peptide (Figure 4(b)). To determine whether the effects of the S5 peptide were recapitulated in primary cells, peritoneal macrophages were harvested from ICR mice and the phosphorylation status of various signaling molecules was investigated by Western blotting. This experiment revealed that the S5 peptide decreased the phosphorylation of c-Jun, p38, and MKK3/6 in LPS-driven signaling (Figure 4(c)). Moreover, the S5 peptide also dramatically blocked the interaction of phospho-p38 with c-Jun and its phosphoform, as revealed by immunoprecipitation and immunoblotting analyses (Figure 4(d)). Based on these results, we conclude that the S5 peptide is capable of preventing AP-1 signaling events. Some of strong anti-inflammatory remedies such as medicinal plant-derived extracts including* Dryopteris crassirhizoma* and* Archidendron clypearia* and their individual components including andrographolide, ginsenosides, curcumol, and lutein are known to suppress AP-1 function in their pharmacological actions [34–38], suggesting that AP-1 could play a critical role in inflammation-regulatory functions. Due to their size and charge, some peptides are regarded as one of membrane nonpermeable biomaterials. In contrast, most hydrophobic small chemicals are much assessable to be penetrated into the membrane and display higher possibility to directly interact with cytosolic target enzymes. However, chemical drugs showing effective inhibition property toward important signaling enzymes are faced to have another side effect causing nonspecific toxicity. In this respect, peptide drugs are considered to be favorable in terms of drug development, due to their selectivity. Previously, we reported that peptide K5 is able to suppress TNF-*α* production in LPS-treated RAW264.7 cells up to 48% at 100 *μ*g/mL [28]. On the other hand, chemically modified S5 peptide having free OH groups exhibited stronger activity (80% inhibition at 100 *μ*g/mL) under the same conditions (Figure 1(b)), implying that membrane permeability of peptide S5 could be more improved than K5. Nonetheless, the fact that K5 was revealed to block the release of TNF-*α* and PGE_2_ between 50 to 100 *μ*g/mL [28] is still raising a possibility that some of peptide receptor such as PEPT1, OATP2BA, or OARP1B3 [39, 40] could contribute to the uptake of peptides K5 and S5 into cytoplasm. The point that S5 peptide exhibits higher anti-inflammatory property under improved membrane permeability seems to impose S5 peptide to be developed as anti-inflammatory peptide drug. Indeed, some peptides such as GLP and its analogs, extracellular matrix protein-derived peptides [41], and integrin-binding N-terminal peptides [42] are closed to be developed as antitumorigenic remedy, although the therapeutic use of these peptides is still limited due to high cost, poor stability, and mediocre pharmacokinetics [18]. In cases of the S5 and K5 peptides, these are known to be self-assembling nanopeptides forming a hydrogel applied for drug delivery system and have been shown to be nonimmunogenic and noninvasive in the filling of cavities [15, 18, 43]. According to our results, these peptides are found to have anti-inflammatory properties which can give additional benefits when they are used as carrier to deliver immunomodulatory drugs. Since the S5 peptide itself suppresses events in the TLR4 signaling pathway, including activation of MAPK and AP-1, it may exert a synergetic effect if carrier molecules are NF-*κ*B-targeted anti-inflammatory drugs.

**Figure 5:**
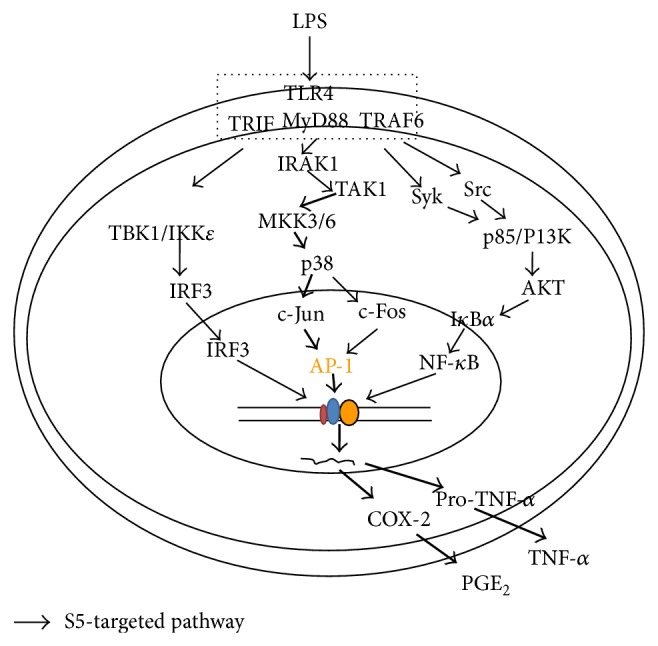
Anti-inflammatory effects of the S5 peptide on signaling pathways.

To sum up these results, here we showed that the S5 peptide dampens various components of the TLR4-mediated inflammatory response, such as the production of PGE_2_ and TNF-*α*. In addition, the S5 peptide reduces AP-1 signaling by preventing the phosphorylation of MKK3/6 and TAK1, as summarized in Figure 5. Since the S5 peptide is a self-assembling peptide that could be used for drug delivery, it is possible that the S5 peptide could be loaded with therapeutics that target AP-1 or other compounds that synergize with inflammatory molecules. Future studies will test the efficacy of the S5 peptide as a drug carrier and as an anti-inflammatory agent in an animal model. These experiments have the potential to contribute to the development of an efficacious and novel drug delivery system.

## Figures and Tables

**Table 1:** Sequences of primers used in real-time PCR analysis.

Gene		Primer sequence
*TNF*-*α*	F	5′-TGCCTATGTCTCAGCCTCTT-3′
	R	5′-GAGGCCATTTGGGAACTTCT-3′

*COX*-*2 *	F	5′-CACTACATCCTGACCCACTT-3′
	R	5′-ATGCTCCTGCTTGAGTATGT-3′

*IL*-*1β*	F	5′-TAGAGCTGCTGGCCTTGTTA-3′
	R	5′-ACCTGTAAAGGCTTCTCGGA-3′

*GAPDH *	F	5′-CACTCACGGCAAATTCAACGGCAC-3′
	R	5′-GACTCCACGACATACTCAGCAC-3′

## References

[1] Yang W. S., Lee J., Kim T. W. (2012). Src/NF-*κ*B-targeted inhibition of LPS-induced macrophage activation and dextran sodium sulphate-induced colitis by *Archidendron clypearia* methanol extract. *Journal of Ethnopharmacology*.

[2] Yu T., Ahn H. M., Shen T. (2011). Anti-inflammatory activity of ethanol extract derived from *Phaseolus angularis* beans. *Journal of Ethnopharmacology*.

[3] Yu T., Lee J., Lee Y. G. (2010). In vitro and in vivo anti-inflammatory effects of ethanol extract from Acer tegmentosum. *Journal of Ethnopharmacology*.

[4] Sekine Y., Yumioka T., Yamamoto T. (2006). Modulation of TLR4 signaling by a novel adaptor protein signal-transducing adaptor protein-2 in macrophages. *Journal of Immunology*.

[5] Ayele Y., Kim J.-A., Park E. (2013). A methanol extract of *Adansonia digitata* L. leaves inhibits pro-inflammatory iNOS possibly via the inhibition of NF-*κ*B activation. *Biomolecules and Therapeutics*.

[6] Kim D. H., Chung J. H., Yoon J. S. (2013). Ginsenoside Rd inhibits the expressions of iNOS and COX-2 by suppressing NF-*κ*B in LPS-stimulated RAW264.7 cells and mouse liver. *Journal of Ginseng Research*.

[7] Stuhlmüller B., Ungethüm U., Scholze S. (2000). Identification of known and novel genes in activated monocytes from patients with rheumatoid arthritis. *Arthritis and Rheumatism*.

[8] Ko H. J., Kwon O. S., Jin J. H., Son K. H., Kim H. P. (2013). Inhibition of experimental systemic inflammation (septic inflammation) and chronic bronchitis by new phytoformula BL containing *Broussonetia papyrifera* and *Lonicera japonica*. *Biomolecules and Therapeutics*.

[9] Burmester G. R., Stuhlmüller B., Keyszer G., Kinne R. W. (1997). Mononuclear phagocytes and rheumatoid synovitis. Mastermind or workhorse in arthritis?. *Arthritis & Rheumatism*.

[10] Bresnihan B. (1999). Pathogenesis of joint damage in rheumatoid arthritis. *Journal of Rheumatology*.

[11] Gracie J. A., Forsey R. J., Chan W. L. (1999). A proinflammatory role for IL-18 in rheumatoid arthritis. *Journal of Clinical Investigation*.

[12] Zhang S. (2002). Emerging biological materials through molecular self-assembly. *Biotechnology Advances*.

[13] Zhang S. (2003). Fabrication of novel biomaterials through molecular self-assembly. *Nature Biotechnology*.

[14] Keyes-Baig C., Duhamel J., Fung S.-Y., Bezaire J., Chen P. (2004). Self-assembling peptide as a potential carrier of hydrophobic compounds. *Journal of the American Chemical Society*.

[15] Nagai Y., Unsworth L. D., Koutsopoulos S., Zhang S. (2006). Slow release of molecules in self-assembling peptide nanofiber scaffold. *Journal of Controlled Release*.

[16] Tysseling-Mattiace V. M., Sahni V., Niece K. L. (2008). Self-assembling nanofibers inhibit glial scar formation and promote axon elongation after spinal cord injury. *The Journal of Neuroscience*.

[17] Garreta E., Gasset D., Semino C., Borrós S. (2007). Fabrication of a three-dimensional nanostructured biomaterial for tissue engineering of bone. *Biomolecular Engineering*.

[18] Koutsopouios S., Unsworth L. D., Nagai Y., Zhang S. (2009). Controlled release of functional proteins through designer self-assembling peptide nanofiber hydrogel scaffold. *Proceedings of the National Academy of Sciences of the United States of America*.

[19] Kabiri M., Bushnak I., McDermot M. T., Unsworth L. D. (2013). Toward a mechanistic understanding of ionic self-complementary peptide self-assembly: role of water molecules and ions. *Biomacromolecules*.

[20] Cho J. Y., Baik K. U., Jung J. H., Park M. H. (2000). In vitro anti-inflammatory effects of cynaropicrin, a sesquiterpene lactone, from Saussurea lappa. *European Journal of Pharmacology*.

[21] Pauwels R., Balzarini J., Baba M. (1988). Rapid and automated tetrazolium-based colorimetric assay for the detection of anti-HIV compounds. *Journal of Virological Methods*.

[22] Kim B. J. (2013). Involvement of melastatin type transient receptor potential 7 channels in ginsenoside Rd-induced apoptosis in gastric and breast cancer cells. *Journal of Ginseng Research*.

[23] Yu T., Lee Y. J., Yang H. M. (2011). Inhibitory effect of Sanguisorba officinalis ethanol extract on NO and PGE_2_ production is mediated by suppression of NF-*κ*B and AP-1 activation signaling cascade. *Journal of Ethnopharmacology*.

[24] Kwon J., Kim S., Shim S., Choi D. S., Kim J. H., Kwon Y. B. (2011). Modulation of LPS-stimulated astroglial activation by ginseng total saponins. *Journal of Ginseng Research*.

[25] Kim J. Y., Lee Y. G., Kim M.-Y. (2010). Src-mediated regulation of inflammatory responses by actin polymerization. *Biochemical Pharmacology*.

[26] Lee Y. G., Lee W. M., Kim J. Y. (2008). Src kinase-targeted anti-inflammatory activity of davallialactone from Inonotus xeranticus in lipopolysaccharide-activated RAW264.7 cells. *British Journal of Pharmacology*.

[27] Zhang S. (2008). Designer self-assembling Peptide nanofiber scaffolds for study of 3-d cell biology and beyond. *Advances in Cancer Research*.

[28] Yang W. S., Park Y. C., Kim J. H. (2012). Nanostructured, self-assembling peptide K5 blocks TNF*α*- and PGE_2_ production by suppression of the AP-1/p38 pathway. *Mediators of Inflammation*.

[29] Hartmann E. K., Ziebart A., Thomas R. (2015). Inhalation therapy with the synthetic TIP-like peptide AP318 attenuates pulmonary inflammation in a porcine sepsis model. *BMC Pulmonary Medicine*.

[30] Altmann K., Wutkowski A., Klempt M., Clawin-Rädecker I., Meisel H., Lorenzen P. C. (2015). Generation and identification of anti-inflammatory peptides from bovine *β*-casein using enzyme preparations from cod and hog. *Journal of the Science of Food and Agriculture*.

[31] Yoo J. H., Ho S., Tran D. H. (2015). Antifibrogenic effects of the antimicrobial peptide cathelicidin in murine colitis-associated fibrosis. *Cellular and Molecular Gastroenterology and Hepatology*.

[32] Yang Y., Kim S. C., Yu T. (2014). Functional roles of p38 mitogen-activated protein kinase in macrophage-mediated inflammatory responses. *Mediators of Inflammation*.

[33] Sakurai H. (2012). Targeting of TAK1 in inflammatory disorders and cancer. *Trends in Pharmacological Sciences*.

[34] Shen T., Yang W. S., Yi Y.-S. (2013). AP-1/IRF-3 targeted anti-inflammatory activity of andrographolide isolated from *Andrographis paniculata*. *Evidence-Based Complementary and Alternative Medicine*.

[35] Oh J., Kim J. H., Park J. G. (2013). Radical scavenging activity-based and AP-1-targeted anti-inflammatory effects of lutein in macrophage-like and skin keratinocytic cells. *Mediators of Inflammation*.

[36] Yang Y., Lee G. J., Yoon D. H. (2013). ERK1- and TBK1-targeted anti-inflammatory activity of an ethanol extract of *Dryopteris crassirhizoma*. *Journal of Ethnopharmacology*.

[37] Chen X., Zong C., Gao Y. (2014). Curcumol exhibits anti-inflammatory properties by interfering with the JNK-mediated AP-1 pathway in lipopolysaccharide-activated RAW264.7 cells. *European Journal of Pharmacology*.

[38] Yang W. S., Jeong D., Nam G. (2013). AP-1 pathway-targeted inhibition of inflammatory responses in LPS-treated macrophages and EtOH/HCl-treated stomach by Archidendron clypearia methanol extract. *Journal of Ethnopharmacology*.

[39] Taub M. E., Mease K., Sane R. S. (2011). Digoxin is not a substrate for organic anion-transporting polypeptide transporters OATP1A2, OATP1B1, OATP1B3, and OATP2B1 but is a substrate for a sodium-dependent transporter expressed in HEK293 cells. *Drug Metabolism and Disposition*.

[40] Tamai I. (2012). Oral drug delivery utilizing intestinal OATP transporters. *Advanced Drug Delivery Reviews*.

[41] Kim S. D., Lee H. Y., Shim J. W. (2011). Activation of CXCR2 by extracellular matrix degradation product acetylated pro-gly-pro has therapeutic effects against sepsis. *American Journal of Respiratory and Critical Care Medicine*.

[42] Seo D.-W., Saxinger W. C., Guedez L., Cantelmo A. R., Albini A., Stetler-Stevenson W. G. (2011). An integrin-binding N-terminal peptide region of TIMP-2 retains potent angio-inhibitory and anti-tumorigenic activity in vivo. *Peptides*.

[43] Gelain F., Unsworth L. D., Zhang S. (2010). Slow and sustained release of active cytokines from self-assembling peptide scaffolds. *Journal of Controlled Release*.

